# Gastrointestinal stromal tumor of the stomach with lymph node metastasis

**DOI:** 10.1186/1477-7819-6-97

**Published:** 2008-09-05

**Authors:** Aras Emre Canda, Yucel Ozsoy, Olcay Ak Nalbant, Ozgul Sagol

**Affiliations:** 1 Department of Surgery, Manisa State Hospital, Manisa, Turkey; 2 Department of Pathology, Manisa State Hospital, Manisa, Turkey; 3 Department of Pathology, Dokuz Eylul University School of Medicine, Izmir, Turkey

## Abstract

**Background:**

Lymph node (LN) metastasis of gastrointestinal stromal tumors (GIST) is unusual. Unlike gastric adenocarcinomas, routine lymphadenectomy is not recommended unless there is no suspicion for LN metastasis. Herein, we report a case of GIST of the stomach with LN metastasis treated with distal gastrectomy with perigastric LN dissection followed by adjuvant imatinib therapy.

**Case presentation:**

A 32-year-old female presented with anemia. Diagnostic investigations including thoracoabdominopelvic computed tomography (CT) scan and gastroscopy revealed a 8 cm gastric antral submucosal tumor without any metastasis. Enlarged periantral LNs were detected during laparotomy and patient underwent distal gastrectomy with *en bloc *perigastric LN dissection. Pathologic investigation revealed antral stromal tumor with high mitotic and Ki-67 index. Lymph node metastasis was observed in 7 of 12 resected perigastirc nodes. Immunohistochemically, tumor cells were positive for CD117. She was diagnosed as high grade gastric GIST due to the presence of LN metastasis, large tumor size and unfavorable histopathologic features thus underwent adjuvant imatinib treatment (400 mg, daily). No recurrence or metastasis has been detected during a 12-month of postoperative follow-up.

**Conclusion:**

Surgery remains the mainstay of treatment in patients with localized, resectable GISTs. Although lymphatic metastasis rarely occurs in patients with GIST, LN dissection should be considered for patients with any suspicion of nodal metastasis. Adjuvant imatinib treatment is recommended according to the well defined prognostic factors.

## Background

Gastrointestinal stromal tumor (GIST) is the most common mesenchymal tumor of the gastrointestinal tract. They most commonly arise from the stomach; which account for ~1% of gastric malignancies [1]. Their origin has been proposed to be the intestinal cells of Cajal [2]. The mainstay of primary treatment for GIST is R0 resection. Unlike gastric adenocarcinomas, routine lymphadenectomy is not recommended unless there is no suspicion of intraoperative lymph node (LN) metastasis. Approximately 95% of GISTs express mutation in the *C-KIT *proto-oncogen [3]. A tyrosine kinase inhibitor, Imatinib mesylate (Glivec^®^; Novartis Pharma, Istanbul, Türkiye) which blocks KIT proteins is the main agent for targeted adjuvant and neoadjuvant treatment as well as used for palliation. Risk assessment after resection determines the need for adjuvant imatinib treatment. Currently, main indications for adjuvant imatinib treatment are unresectable or metastatic disease [4]. Herein, we report a case of GIST of the stomach with LN metastasis and discussed its management and follow-up.

## Case presentation

**Figure 1:**
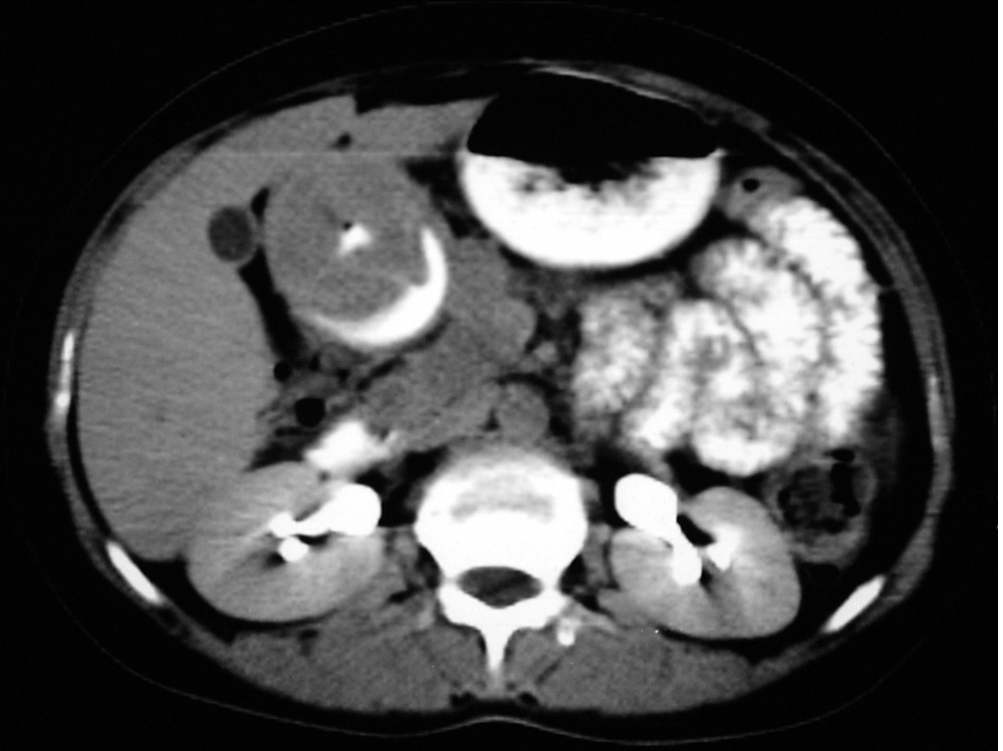
Abdominal CT scan: axial view showing an 8 cm in size antral intramural mass.

A 32-year-old female with anemia was referred to our hospital. Her past medical history was insignificant. Gastroscopy demonstrated an antral submucosal tumor. Thoracoabdominopelvic computed tomography (CT) scan showed an 8 cm intramural mass with no distant metastasis (Figure 1). At laparotomy, few enlarged periantral LNs around the tumor reaching up to 1 cm were observed. Distal gastrectomy with *en bloc *perigastric LN dissection was performed. Postoperative course of the patient was uneventful.

**Figure 2:**
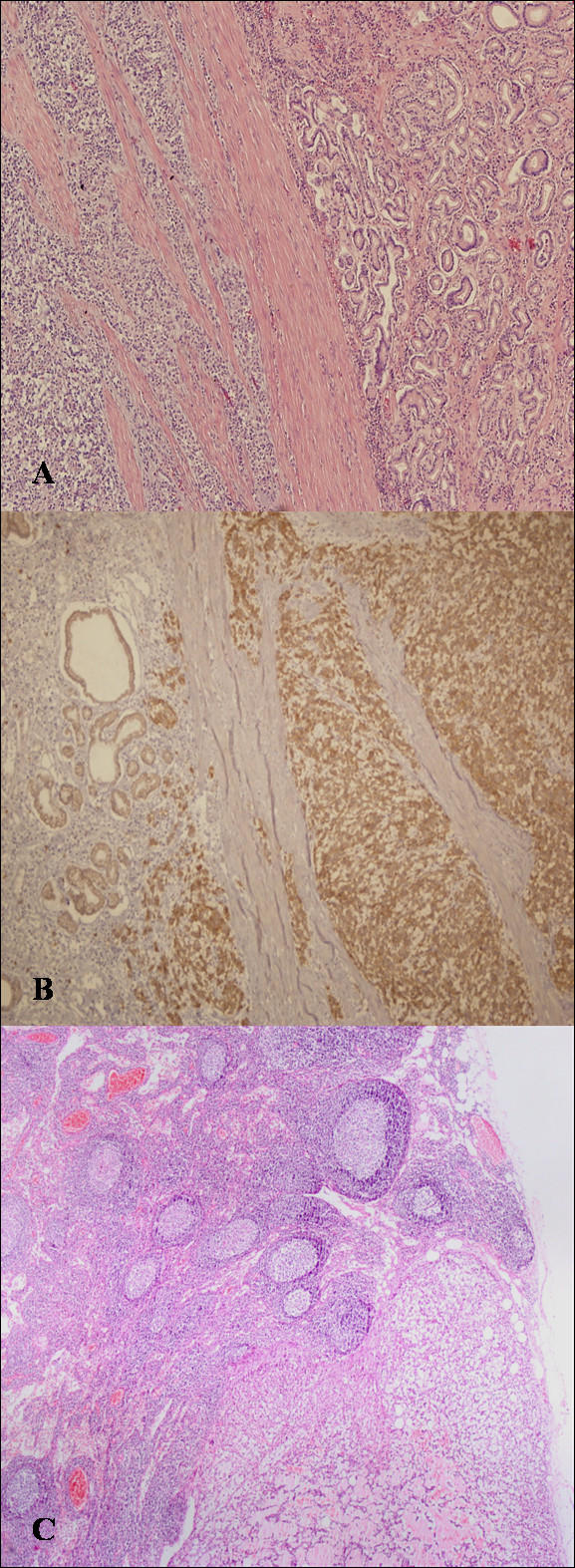
**The histopathological examination of the tumor.A: **A photomicrograph of the tumor showing epithelioid cells with prominent pleomorphism; mitotic index was 25 mitoses/50 hpf and MiB1 (Ki-67) index was higher than 10% (H&E stain). **B: **Immunoreactivity for CD117 of the tumor cells. **C: **A photomicrography of a lymph node metastasis (H&E stain).

Histopathological examination showed an antral stromal tumor which was 8 × 8 × 4 cm in size. Mitotic index was 25 mitoses/50 high-power fields (hpf) and MiB1 (Ki-67) index was higher than 10% (Figure 2a). No necrosis and infiltration to adjacent structures was observed. Immunohistochemically, tumor cells were positive for CD117 (+++) and CD34 (+++); negative for desmin and S-100 (Figure 2b). Lymph node metastasis was observed in 7 of 12 resected perigastirc nodes (Figure 2c).

She was diagnosed as high grade gastric GIST due to the presence of LN metastasis, large tumor size and unfavorable histopathological features (high mitotic index and Ki-67 index). Therefore, adjuvant imatinib treatment (Glivec^® ^400 mg, daily) was initiated and has been continued to date. The drug was well tolerated by the patient and no adverse effect was observed. No recurrence or metastasis has been detected during a 12-month postoperative follow-up.

## Discussion

GISTs are distinctive subgroup of gastrointestinal mesenchymal tumors which express CD117 or platelet derived growth factor receptor alpha (PDGFRA) [3,5]. Due to the recent advances in immunohistochemical and molecular techniques, its diagnostic incidence has been increased. Most of the patients with GIST are symptomatic and bleeding due to mucosal ulceration is the most common symptom [6].

Surgery remains the mainstay of treatment in patients with localized, resectable GISTs. The principle of surgery for GISTs is R0 resection of the tumor. Tumor rupture or R1 resection of the primary tumor has a negative impact on disease free survival [7]. Aparicio *et al*. [8] reported lower local recurrence rates with segmental resection of the stomach compared to wedge gastric resection even in patients whom R0 resection was obtained. Lymphatic metastasis rarely occurs (0–3.4%) in patients with GIST [1,9,10]. Special care was taken during the histopathological examination for differentiation of nodal metastasis from peritoneal dissemination of the tumor. Although there is limited experience with management of GISTs with LN metastasis; LN dissection should be considered for patients with any suspicion of nodal metastasis. In our patient, because the enlarged LNs were located at the periantral region, we performed a limited lymphatic dissection (stations 3–9). This surgical approach documented synchronous nodal metastasis status thus contributed for decision of the adjuvant treatment planning. The postoperative course of the patient was uneventful.

Reported recurrence rates of 17–21% and 5-year survival rates of 48–70% even in patients with resectable GIST emerges the need for an adjuvant treatment [8,11-18]. The American Collage of Surgeons Oncology Group (ACOSOG) Z9001 trial is a randomized trial of imatinib versus placebo administered for one year following complete resection of a primary GIST which demonstrated a significant improvement in recurrence free survival with imatinib [19].

Currently, there is no accepted staging system for GISTs. Tumor size, location, mitotic rate, *C-KIT *and PDGFRA genotype are the major determinants of malignant potential of the tumor which have significant impact on prognosis [20-22]. A practical grading system for GIST after surgical resection was proposed by Bucher *et al*. [22] including 5 minor (tumor size ≥5 cm, mitotic index ≥5 mitoses/50 hpf, presence of necrosis, infiltration of adjacent structures, and MiB1 index > 10%) and two major (presence of LN invasion and/or metastasis) criteria. Tumors having less than four minor criteria were classified as low grade GIST and tumors having four or five minor criteria or one major criterion were classified as high grade GIST.

It is not well established which patients will benefit from adjuvant imatinib treatment and the duration of treatment after complete resection of the primary GIST. Several recent trials have directed efforts to determine which patients may be more likely to benefit from adjuvant imatinib treatment and its duration. Bucher *et al*. [22] showed a correlation between the staging system and disease free survival and patient survival after primary surgery. They propose adjuvant imatinib treatment for high grade GIST patients. European Organization for Research and Treatment of Cancer (EORTC) 62024 phase-III ongoing trial randomizes patients with intermediate- and high-risk GIST in whom complete macroscopic resection achieved for treatment with imatinib (400 mg, daily) *vs. *placebo for 2 years. In ACOSOG Z9001 phase-III trial, adjuvant treatment is recommended for at least 12 months although the optimal duration has not yet been determined [19].

## Conclusion

Surgery remains the mainstay of treatment in patients with localized, resectable GISTs. Although lymphatic metastasis rarely occurs in patients with GIST, LN dissection should be considered for patients with suspicion of nodal metastasis. Due to the presence of three minor and one major unfavorable prognostic factors, we considered our patient as high grade GIST thus initiated adjuvant imatinib treatment. We will consider the duration of imatinib treatment according to drug's tolerability and patient's clinical outcome and due to future scientific evidence.

## List of abbreviations

GIST: Gastrointestinal stromal tumors; CT: Computed tomography; hpf: High-power fields; PDGFRA: Platelet derived growth factor receptor alpha; ACOSOG: American Collage of Surgeons Oncology Group; EORTC: European Organization for Research and Treatment of Cancer.

## Competing interests

The authors declare that they have no competing interests.

## Authors' contributions

AEC drafted the manuscript, YO helped in preparation and editing of the manuscript, ON wrote the pathological part of the manuscript and OS performed the IHC and contributed the IHC part of the manuscript and photomicrographs. All authors read and approved the final manuscript.

## Consent

Written consent was obtained from the patient for publication of this study.
